# A Performance Analysis of Security Protocols for Distributed Measurement Systems Based on Internet of Things with Constrained Hardware and Open Source Infrastructures

**DOI:** 10.3390/s24092781

**Published:** 2024-04-26

**Authors:** Antonio Francesco Gentile, Davide Macrì, Domenico Luca Carnì, Emilio Greco, Francesco Lamonaca

**Affiliations:** 1 Institute for High-Performance Computing and Networking (ICAR), National Research Council of Italy (CNR), Via P. Bucci 8/9C, 87036 Rende, Italy; antoniofrancesco.gentile@icar.cnr.it (A.F.G.); davide.macri@icar.cnr.it (D.M.); emilio.greco@icar.cnr.it (E.G.); 2 Department of Computer Engineering, Modeling, Electonics and Systems Engineering (DIMES), University of Calabria, Via P. Bucci 39/c, 87036 Rende, Italy; dl.carni@dimes.unical.it; 3 Institute of Nanotechnology (CNRNANOTEC), National Research Council of Italy (CNR), Via P. Bucci 31C, 87036 Rende, Italy

**Keywords:** distributed measurement systems, DMS, MQTT, MQTTs, Mosquitto, Raspberry Pi 4, Linux, OpenWrt, IoT, TLSv1.2, TLSv1.3, SSL

## Abstract

The widespread adoption of Internet of Things (IoT) devices in home, industrial, and business environments has made available the deployment of innovative distributed measurement systems (DMS). This paper takes into account constrained hardware and a security-oriented virtual local area network (VLAN) approach that utilizes local message queuing telemetry transport (MQTT) brokers, transport layer security (TLS) tunnels for local sensor data, and secure socket layer (SSL) tunnels to transmit TLS-encrypted data to a cloud-based central broker. On the other hand, the recent literature has shown a correlated exponential increase in cyber attacks, mainly devoted to destroying critical infrastructure and creating hazards or retrieving sensitive data about individuals, industrial or business companies, and many other entities. Much progress has been made to develop security protocols and guarantee quality of service (QoS), but they are prone to reducing the network throughput. From a measurement science perspective, lower throughput can lead to a reduced frequency with which the phenomena can be observed, generating, again, misevaluation. This paper does not give a new approach to protect measurement data but tests the network performance of the typically used ones that can run on constrained hardware. This is a more general scenario typical for IoT-based DMS. The proposal takes into account a security-oriented VLAN approach for hardware-constrained solutions. Since it is a worst-case scenario, this permits the generalization of the achieved results. In particular, in the paper, all OpenSSL cipher suites are considered for compatibility with the Mosquitto server. The most used key metrics are evaluated for each cipher suite and QoS level, such as the total ratio, total runtime, average runtime, message time, average bandwidth, and total bandwidth. Numerical and experimental results confirm the proposal’s effectiveness in foreseeing the minimum network throughput concerning the selected QoS and security. Operating systems yield diverse performance metric values based on various configurations. The primary objective is identifying algorithms to ensure suitable data transmission and encryption ratios. Another aim is to explore algorithms that ensure wider compatibility with existing infrastructures supporting MQTT technology, facilitating secure connections for geographically dispersed DMS IoT networks, particularly in challenging environments like suburban or rural areas. Additionally, leveraging open firmware on constrained devices compatible with various MQTT protocols enables the customization of the software components, a crucial necessity for DMS.

## 1. Introduction

With the proliferation of Internet of Things (IoT) devices, safeguarding sensitive data has become paramount. As highlighted by the recent literature [1], many of these data are measurements, and the IoT paradigm is the basis of modern distributed measurement systems (DMSs), used for the monitoring of many heterogeneous physical quantities, crucial for wellness [2,3], industrial automation, education, power generation and distribution, smart agriculture, and farming [4,5], but also for the safety of people [6,7,8] and structures [9]. The cyber security company Check Point Research has reported a significant surge in cyber attacks targeting IoT devices during the current calendar year. In the initial two months of 2023, there was a 41% rise in the weekly frequency of IoT device attacks per organization, in comparison to the figures from 2022, with the education and research sector experiencing the most pronounced rise in the attack frequency. Throughout the initial two months of 2023, approximately 54% of organizations were targeted by these attack attempts weekly, averaging almost 60 attacks per organization per week specifically aimed at IoT devices. This represents a 41% increase compared to 2022, and it is more than three times the number of attacks observed two years ago [10,11].

For completeness, within the scope of cyber security, commonly used security measures are the zero trust architecture (ZTA), machine learning and AI-based security, hardware-based security, and regulatory compliance and standards. These approaches are not suitable to be implemented on commercially constrained hardware, which is a more common scenario, especially for IoT-based distributed measurement systems (DMS). In these cases, as suggested by [12], the VLAN approach is recommended.

This phenomenon is associated with the expansion of connected technology, particularly in home automation and smart buildings [13]. Four key factors contributing to this surge in attacks can be identified:

- The rapid expansion of Internet-connected IoT devices that can be controlled remotely;
- Low-profit margins in the home automation component market, which restrict investments in security;
- The significance of the speed to market, driving companies to compete for the first-mover advantage;
- Limited end-user awareness of cyber security, often prioritizing cost over security.

As a consequence, many approaches are available to guarantee quality of service [14] and safety [15]. Still, these solutions can reduce the network throughput; in other words, they can delay the exchange of measurement information. This can provoke a reduction in the frequency with which the physical phenomena are observed and, consequently, can complicate its evaluation [16]. The consequence may be the vulnerability of the whole system and its malfunctioning.

Since the IoT devices and connection modalities are strongly heterogeneous, this paper takes into account constrained hardware and a security-oriented virtual local area network (VLAN) approach that utilizes local message queuing telemetry transport (MQTT) brokers, TLS tunnels for local sensor data, and an SSL tunnel to transmit TLS-encrypted data to a cloud-based central broker. This context can be considered a worst-case scenario, suitable for generalizing the results achieved.

Table 1 illustrates all OpenSSL [17] cipher suites of the TLSv1.2 and TLSv1.3 families that are compatible with MQTT brokers [18] tested and considered secure.

Some cryptographic analyzed suites are recommended by the Agency for Digital Italy (AGID) [19] to offer recommendations regarding security protocols and cipher Suites representing the state of the art. Indeed, due to ongoing technological evolution and the possible discovery of new vulnerabilities, this document is periodically updated and specific security advisories may be issued.

This paper has the following structure: Section 1 shows related works about IoT-secure implementations and analyses presented in the literature; Section 3 presents our proposal; Section 4 presents the architecture and its deployment; Section 5 provides the measurement method for the throughput evaluation; Section 7 presents the experimental results; lastly, Section 8 summarizes the paper with the discussion and conclusions.

## 2. Related Works

This section describes related works about IoT-secure implementations and analyses present in the literature.

The paper [12] discusses how all existing RTE networks leverage the foundational aspects of Ethernet, employing protocol tactics such as the strategic utilization of virtual LAN (VLAN) prioritization. Some networks even incorporate non-standard data link layers to integrate real-time functionalities into a network infrastructure that inherently lacks real-time support.

Instead, the articles [3,6] examine the influence of the TLS protocol on the security and performance of the MQTT protocol. The security assessment delves into authentication, data privacy, and data integrity. Concurrently, the performance evaluation focuses on the time consumption and the volume of data exchanged between the MQTT client and broker. Additionally, power consumption is assessed for both scenarios involving MQTT.

Among the literature dedicated to the IoT, several studies have delved into the functionality of MQTT, with a specific focus on the broker node. In research [20], the authors tested the performance of several MQTT broker implementations using a physical platform based on a Raspberry Pi. However, the paper lacks a comparative assessment of the performance across different transport protocols, which could have provided more insights into the strengths and weaknesses of each protocol. It also lacks consideration of how different protocols perform regarding speed, reliability, efficiency, and security and how they handle different types of traffic and network conditions. Future research could explore this aspect further. Gammes et al. [21] conducted a study on the performance of Mosquitto, one of the most commonly used MQTT broker implementations, both under normal conditions and in the presence of denial of service (DoS) attacks. Taking a broader perspective, Gheorghe-Pop et al. [22] detailed a benchmark evaluation conducted across various MQTT broker solutions. It is interesting to note that Mishra et al. conducted a study evaluating the efficacy of various MQTT brokers under stress conditions. The researchers found that the Mosquitto implementation outperformed other options across most parameters. It is pertinent to consider how different solutions fare under varying conditions. Koziolek and colleagues conducted a study comparing the performance of three distributed MQTT broker implementations. However, their focus was on usability, CPU performance, reliability, and related aspects. They published their findings in a paper titled ‘Comparison of Distributed MQTT Broker Implementations’ [23]. The performance evaluation of application layer protocols for IoT and IIoT has garnered attention in various works. MQTT is a standout solution due to its widespread adoption and extensive analysis. For instance, Ebleme et al. [24] assessed MQTT’s behavior regarding the delay, throughput, and energy consumption. They utilized Arduino-based nodes as IoT devices for their investigation. In a different context, Katsikeas et al. [25] determined MQTT’s suitability for industrial scenarios, emphasizing its lightweight nature. Their evaluation focused on data security, comprehensively addressing potential security issues, and examined networking features in a real IIoT scenario, utilizing a wind park for their study.

Michaelides et al. delved into the security aspects of MQTT in [26], although they did not specifically assess the delay. Like our approach, they employed a Raspberry Pi in their testbed and characterized the energy consumption. Silva et al. conducted a study wherein they compared and evaluated different IoT communication protocols, such as MQTT, CoAP [27], and Open Platform Communications Unified. Their findings were presented in [28].

Pohl et al. [29] analyzed several IoT protocols, including the Advanced Message Queuing Protocol (AMQP), MQTT, and Extensible Messaging and Presence Protocol (XMPP), using a three-tier testbed. The study yielded some interesting results. According to their findings, MQTT outperformed AMQP and XMPP in various categories, like latency, throughput, bandwidth, and reliability.

Seoane et al. conducted a study that compared the performance of CoAP and MQTT. They used a real testbed and emulation techniques for different channel conditions. To modify the loss rate, they employed the NetEm application. One can find more details about their research in [30].

In their study, Ferrari et al. [31] integrated the Internet of Things (IoT) paradigm into the Industry 4.0 framework. They aimed to monitor the data generated by sensing devices and process them using cloud-based solutions. Considering different scenarios, the authors evaluated the round-trip latency estimation for data transfer between IoT devices and the cloud.

Kenitar and colleagues researched latency estimations and data transmission from the edge to the cloud, as described in their paper [32]. According to the paper, the authors selected MQTT as their data delivery solution.

By analyzing the recent literature, the network performance’s importance in throughput and safety for the development of a distributed measurement system arises. One of the first papers highlighting this important matter appeared in 2017, authored by A. Flammini et al. [16].

Interestingly, the authors noted the similarities between IoT systems and DMS yet also pointed out that IoT systems have never been fully evaluated in terms of DMS requirements like uncertainty and timestamps. This raises important questions for instrumentation and measurement scientists regarding the use of new IoT technologies to replace specialized DMSs. Can the newer IoT technologies deliver the same level of measurement performance? In the paper, the authors suggest that low-power wide area networks (LPWANs) might be a feasible solution for large-scale DMSs.

Research that aims to characterize performance indicators related to time is crucial for distributed systems. The experimental findings demonstrate that low-cost transceivers can schedule frame transmissions with a standard uncertainty of less than 3 μs. Furthermore, commercial devices (nodes and packet forwarders) exhibit acceptable long-term clock stability, as indicated by Allan Deviation. These promising results suggest the suitability of LPWANs for applications such as smart metering, smart buildings, and the process industry.

In the study by Renzone [33], the impact of the challenging industrial environment on the transmission capabilities of IoT nodes was considered. A comprehensive measurement campaign was conducted in controlled environments, revealing a decline in performance under various conditions. Importantly, this decline did not compromise the network reliability or harm the electronic components of the sensor nodes. The article’s primary focus was to investigate the behavior of LoRaWAN transmitters when subjected to a broad spectrum of temperature and humidity variations, ranging from very low to extreme levels. Additionally, the study examined the effects of machinery vibrations and the presence of NO, NO_2_, and CO gases as common elements in industrial settings. Tests were meticulously conducted by transmitting signals in diverse environmental settings using a climatic chamber, a purpose-built vibration test bench, and a fume extraction system. This approach allowed precise control over the temperature, humidity, vibration frequency, amplitude, and gas concentrations, creating a controlled experimental environment. Such conditions mirror the challenges in industrial contexts, such as oil and gas fields, where wireless communication technologies are extensively utilized. Consequently, the article contributes valuable insights into the transmission performance degradation resulting from the specified environmental conditions.

In the study by Ferrigno et al. [34], a novel approach is presented to address security concerns related to heart and heart bleed-like attacks.

It is interesting to note that the authors have proposed a measurement method and experimental setup for inline intrusion detection without needing payload decoding. An advantage of this method is that it can be implemented on low-performance general-purpose processing units, making it suitable for the integration of IoT sensor nodes and gateways. The developed system underwent rigorous testing on a real network. The results showed that its performance was comparable to—or, in some cases, better than—that of more resource-intensive machine learning-based methods.

The experiments involved a pair of personal computers (PCs) connected via Ethernet using the IEEE 802.3 protocol, both running Ubuntu 12.04 with OpenSSL version 1.0.1f for heart bleed attacks.

Symmetry in software features ensured unbiased results. The open-source traffic monitoring software CIC-CICFlowMeter [35] was installed on the receiver side, facilitating bidirectional traffic monitoring and the measurement of 83 parameters for each session. Evaluation metrics, including the true positive (TP), true negative (TN), false positive (FP), and false negative (FN), served as the figure of merit for the proposed measurement method. To adapt the method for IoT frameworks, the authors suggested a network configuration involving IoT nodes passing through an IoT gateway for protection against attacks. The gateway, independent of the device types, filtered and verified traffic, acting as a centralized defense mechanism without needing device-specific patching. In their testbed, the authors utilized two vulnerable Ubuntu 12.04 LTS terminals for the attacker. They attacked the IoT node and a Raspberry Pi 4 Model B with Ubuntu 22.04 LTS for the IoT gateway. The gateway, equipped with Python version 3.10, successfully implemented the proposed measurement method for heart bleed and heart bleed-like inline detection, showcasing its effectiveness on low-cost platforms.

The proposed SoD-MQTT solution in [36] is interesting. The authors have developed a novel approach to real-Time distributed MQTT using software-defined networks (SDN). The design and protocols are optimized to reduce the communication delays between brokers and support low-latency applications such as e-health and transportation. The authors evaluated the effectiveness of their approach by comparing it with existing SDN-based MQTT brokers in terms of latency and network utilization. They utilized Mininet-WiFi, a commonly used tool for the emulation of wireless environments and evaluation of new mechanisms/architectures related to SDN technology.

While these studies encompass a broader scope, our paper explicitly compares the performance of various OpenSSL cipher suite protocols employed to support IoT data delivery via MQTT.

For the sake of completeness, it is essential to remember that the first level at which one can operate for data security is the physical one [37]. Physical layer security in IoT networks involves implementing security measures at the lowest layer of the communication stack to protect against eavesdropping, jamming, tampering, and other threats [38,39]. Techniques include signal encryption, RF fingerprinting, jamming detection, physical tamper resistance, channel authentication, transmission power control, and secure localization [38,39,40]. These measures enhance the resilience of IoT networks, ensuring the integrity, confidentiality, and availability of the communication channels and data transmission, but they require one to modify the hardware of the measurement node communication device [37,38,39,40].

In [41], it is shown that organizational security protocols, maintaining email security gateways with tools like the Sender Policy Framework (SPF) and DomainKeys Identified Mail Policy (DIMP), are essential to verify email origins and ensure message integrity during transit. Additionally, endpoint security measures, including antivirus updates and host-based intrusion detection systems (HIDS), are crucial in mitigating socially engineered attacks. However, the literature emphasizes the challenge that organizations face in ensuring that employees possess the necessary professionalism and proficiency to effectively utilize these security tools.

## 3. Proposal

This section presents our comprehensive proposal, which considers a security-oriented virtual local area network (VLAN) strategy and solutions for hardware constraints. By addressing a worst-case scenario, the research endeavors to generalize the achieved results, ensuring applicability across a spectrum of practical scenarios. The focal point of the study involves thoroughly examining all OpenSSL cipher suites in the context of compatibility with the Mosquitto server.

The paper emphasizes the analysis of key performance metrics, including but not limited to the total ratio, total runtime, average runtime, message time, average bandwidth, and total bandwidth. This evaluation is conducted for each cipher suite and quality of service (QoS) level, providing a nuanced understanding of the intricate interplay between security measures and overall system efficiency.

In pursuit of the first goal, the research aims to identify algorithms that can guarantee an optimal data transmission/encryption ratio. This entails meticulously exploring cryptographic methods and transmission protocols to strike a delicate balance between data security and efficient communication in resource-constrained environments.

The second goal involves a comprehensive investigation into algorithms ensuring compatibility with diverse MQTT infrastructures. The research recognizes the challenges that geographically scattered IoT networks pose, particularly in difficult-to-manage suburban or rural environments. The emphasis is on establishing a secure connection system that accommodates the varied infrastructural nuances inherent in such landscapes. Simultaneously, the third goal focuses on implementing open firmware on constrained devices, fostering compatibility with various MQTT protocols. This initiative is designed to enhance the adaptability and interoperability of IoT devices, thereby contributing to creating a secure and standardized communication framework. Exploring open firmware is integral to addressing the dynamic nature of MQTT protocols and ensuring seamless integration with different devices across the IoT ecosystem.

These three interlinked goals collectively form the backbone of the research, aiming to advance the understanding of secure and efficient IoT communication protocols while addressing the practical challenges posed by hardware constraints and diverse network environments.

## 4. Architecture and Its Deployment

This section presents the architecture and its deployment.

The proposal is based on integrating proper components and protocols running on the following open-source architecture.

MQTT (Mosquitto) refers to the Transmission Control Protocol/Internet Protocol (TCP/IP), which is based on a publish–subscribe model operating through a dedicated message broker. It is one of the most widely used protocols in the field of IoT. Cipher suites are combinations of cryptographic algorithms, protocols, and security parameters that determine how data are encrypted and decrypted in secure communications over the Internet. These suites specify the encryption and authentication methods used to establish a secure connection between a client and a server, such as in Hypertext Transfer Protocol Secure (HTTPS) for secure network protocols. A cipher suite typically includes the following components.

- Key Exchange Algorithm: this securely exchanges encryption keys between the client and the server.
- Encryption Algorithm: this algorithm encrypts the data so that unauthorized parties cannot read them.
- Hash Function: a cryptographic hash function ensures data integrity and authenticity.
- Message Authentication Code (MAC) Algorithm: the MAC ensures the integrity of the data by allowing both parties to detect whether the data have been tampered with during transmission.

VLANs serve as a crucial cybersecurity tool in the IoT and DMS. By offering network segmentation, granular access control, and the isolation of critical systems, VLANs effectively reduce the attack surface and limit the paths for potential attackers. They contribute to efficient traffic monitoring, facilitate the containment of vulnerabilities, and optimize resource allocation, which is particularly crucial in managing the substantial data volumes of IoT devices. Moreover, VLANs simplify network management, enhance compliance enforcement, and provide a scalable security solution adaptable to the evolving needs of expanding IoT environments. Overall, VLANs play a vital role in mitigating cyber risks, improving network resilience, and ensuring the secure operation of IoT-based DMSs.

Cipher suites come in various combinations, and their strength and security levels can vary. The choice of a cipher suite depends on the specific security requirements and the capabilities of the communicating parties. More secure cipher suites use stronger encryption algorithms and key exchange methods, while less secure ones may use weaker encryption, which can be susceptible to attacks [42].

When presenting cipher suites, it is essential to underscore their resistance to side-channel attacks (SCAs) alongside the strength of the encryption algorithms. Cipher suites vary in susceptibility to SCAs, which exploit information leakages from cryptographic algorithm implementations. Evaluations of cipher suites should consider their resilience against various SCAs, with some implementations incorporating countermeasures like constant-time algorithms or randomization. This comprehensive approach ensures robust protection against theoretical cryptographic vulnerabilities and practical implementation-level weaknesses [43,44].

To evaluate the achievable DMS network throughput concerning the partially selected QoS and safety protocols, the proposed measurement method considers a general IoT architecture composed of the following components.

- Local MQTT Brokers: Each site, whether residential or business, has its own dedicated MQTT broker responsible for collecting and managing data from local IoT devices.
- Local TLS Tunnels: Data from sensors within each site are encrypted using local TLS tunnels before being sent to the local MQTT broker.
- Tunnel to Cloud Main Broker: An SSL tunnel is employed to securely transmit data from local brokers to a central cloud-based broker responsible for data aggregation and analytics.

**Figure 1:**
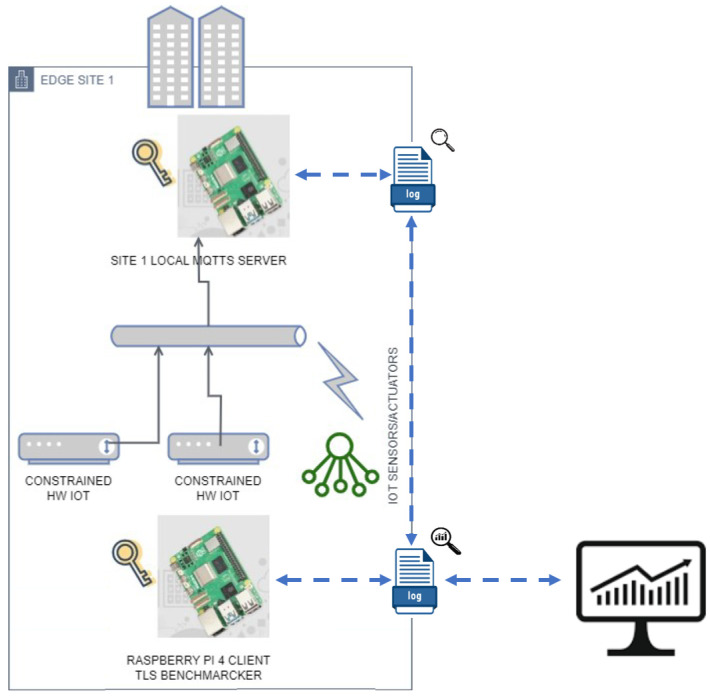
MQTT benchmarking architecture.

Figure 1 shows the deployment of our MQTT benchmarking architecture. The implementation includes the following:

- Integration of MQTT and OpenSSL encryption suites into Raspberry Pi devices;
- Synchronized setup using Network Time Protocol (NTP) for reliable measurements;
- Automation of procedures (sending, recording, etc.) for generation of traces and collection of results;
- Running of tests on different channel technologies, using package mosquitto-client available on Linux utility to measure bandwidth and different loss probabilities.

## 5. Measurement Method for Throughput Evaluation

**Figure 2:**
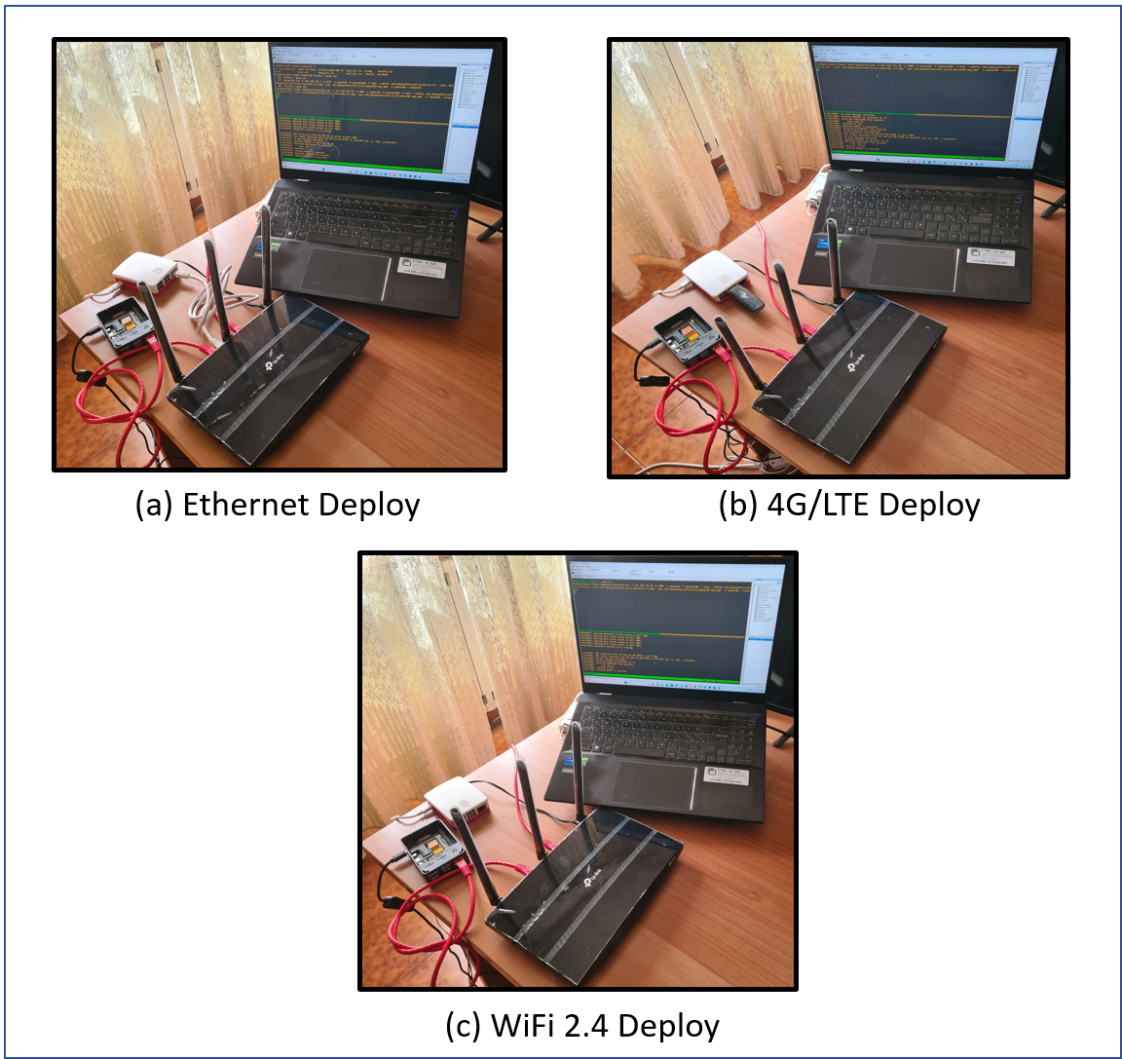
Physical deployment for 4G/LTE, Ethernet, and WiFi testbeds.

This section provides the measurement method for the throughput evaluation. Various factors must be considered for a comprehensive evaluation of the network throughput. The compatibility of all available OpenSSL cipher suites with the MQTT server must be assessed, ensuring that data encryption does not impede communication between IoT devices and brokers. The following pseudocode (in bash scripting) represents the steps followed by the measurement method proposed here. In particular, referring to Figure 1, the throughput between any two nodes of the network is evaluated. Only an MQTT-BRIDGE-type link with related certificates and authentication credentials for clients/servers in different networks is needed. The OpenWrt router acts both as an NTP client and as a local NTP server and is synchronized with the Italian time servers (ntp1.inrim.it, ntp2.inrim.it, time.inrim.it). This ensures that the devices on the network have the same time source. To analyze the performance for each encryption algorithm, we use the software mosquitto-clients package since it permits a more exhaustive analysis concerning its competitors (mqtt-benchmarker [45]; mqttx [46]; mqtt-cli [47]). Then, we compare the results of 1000 message communications varying in terms of the QoS type, encryption algorithm, and TLS version. In Figure 2, the physical deployment for 4G/LTE, Ethernet, and WiFi testbeds is displayed.

The experiments are conducted as follows. A dedicated VLAN is used for IoT networking. The network clock is managed by the router. However, the primary actor is the client. The broker and the client interact via SSH key exchange and both of them have root privileges. The client initiates the main program, which launches 30 consecutive runs of benchmark tests.

At each run, the benchmark tool

- Stops the Mosquitto instance of the broker;
- Queries the server’s OpenSSL version and requests the list of cipher suites before TLSv1.2 and then TLSv1.3 via SSH;
- Creates multiple instances of the broker’s configuration file with the TLS version and cipher suite and executes them one after the other.

For each instance, the benchmark tool

- Uses mosquitto_pub/mosquitto_sub to publish on a specific topic;
- Analyzes the server and client logs;
- Extracts values related to the metrics described in Section 5.1;
- Generates CSV files.

### 5.1. Benchmarking with MQTT Clients and Tools

The software of the previous subsection is used to evaluate the IoT network’s performance. We evaluate the following key metrics when sending a payload of 1 MByte size for each message.

- Total Ratio: The total number of messages sent relative to the messages received, indicating the overall system efficiency.
- Total Runtime (s): The duration of the benchmarking process.
- Average Runtime (s): The average duration of each benchmarking run.
- Message Time Metrics (min, max, mean, and std).
  – Msg time min (ms): The shortest message transit time.
  – Msg time max (ms): The longest message transit time.
  – Msg time mean (ms): The mean of the message transit times.
  – Msg time mean std (ms): The standard deviation of the mean message transit times.
- Average Bandwidth (msg/s): The average number of messages transmitted per second.
- Total Bandwidth (msg/s): The total number of messages transmitted per second during the benchmarking process.

One analyzes the network latency, packet loss, and data integrity to ensure the accuracy and reliability of the sensor data.

Data Confidentiality: Emphasizing the confidentiality of the acquired measurements is crucial, particularly in sensitive areas such as healthcare, financial institutions, and critical structure monitoring. One discusses encryption methods, access controls, and compliance with data protection regulations to ensure that the data remain secure.

## 6. Experimental Testbed

To evaluate the performance of the proposed measurement method in a real scenario, we designed a specific testbed, as shown in Figure 2.

In particular, two Raspberry Pi 4 (8 GB RAM) (https://www.raspberrypi.org, accessed on 14 March 2024) devices are used to implement, respectively, an MQTT client for benchmarking (the white Raspberry) and an MQTT server (the black uncovered Raspberry) from the MQTT benchmarking logical architecture of Figure 1).

**Figure 3:**
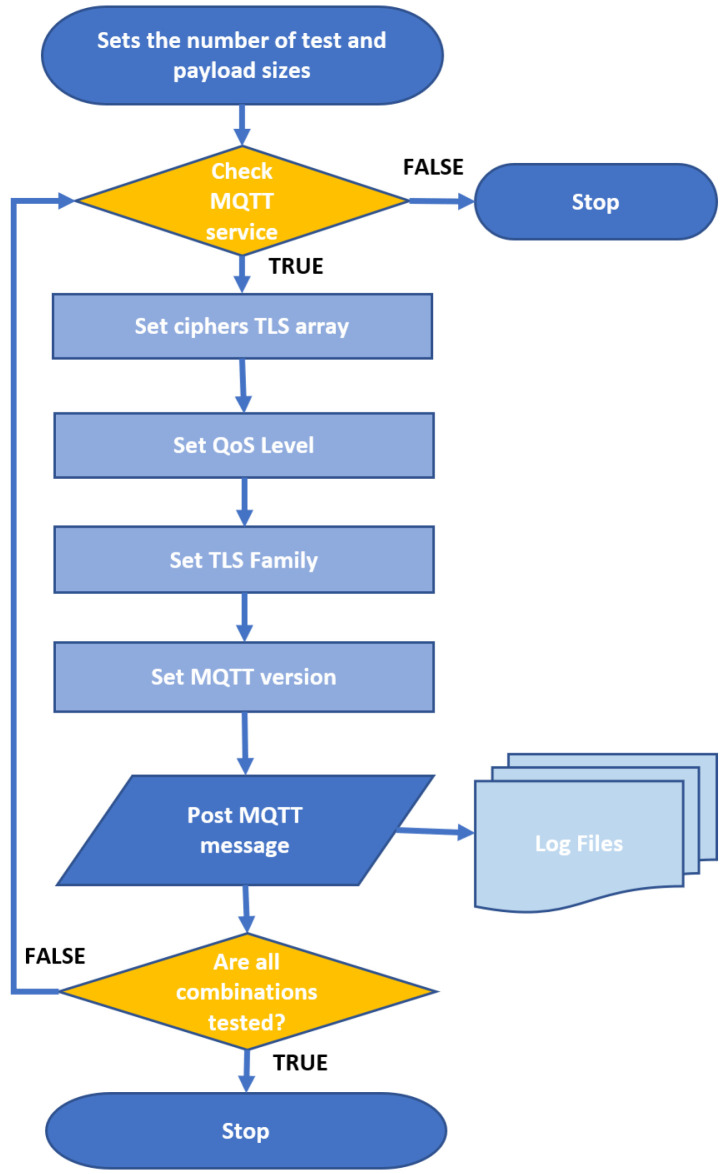
Algorithm flowchart.

Figure 3 illustrates the steps of the algorithm implemented by the two Raspberries. The individual functions ‘mqtt_pub_base-qosX-vY-tlsZ’ used to carry out the tests invoke the software ‘mosquitto_pub/mosquitto_sub’ implemented in the used operative system Debian 12 with Mosquitto version 2.0.11-1.

The algorithm consists of four parts. The first part prepares the benchmarking environment. The second starts the remote MQTT over TLS instances on the broker and dumps the TLS. The third starts the client’s execution for the sending of data. The fourth summarizes the data collection at the end of the session. When forwarding each message, the mqtt_pub_base-qos function collects the forwarding and receiving data within log files through the measurement tool used and connects as a remote syslogger to the mosquitto log file, which is present on the broker.

The reference router is a TP-Link Archer C7 v5 with the Qualcomm Atheros QCA956X ver 1 rev 0 architecture and OpenWrt version 22.03.5.

The tests were conducted on three types of data connections:

- Wired cable (1 Gbps)—Figure 2a;
- 4G/LTE (for connections from rural areas)—Figure 2b;
- Wireless 2.4 GHz (for maximum sensor backward compatibility)—Figure 2c.

For each available cipher suite (regarding the OpenSSL 1.x libraries), a command is sent via SSH (Secure Shell) to initiate the remote broker from the client to the server. At this point, the server will listen on the designated port (in our case, 1866/TCP) with the selected cipher suite one at a time. The client will check if the remote port responds and establish a connection. The ‘mqtt-benchmark’ software, written in Golang, is launched to perform 10 transmissions from 10 datasets for 10 dummy clients, totaling 1000 transmissions. Due to security restrictions in the TLS version, the considered cipher suites are 1.2 and 1.3.

**Figure 4:**
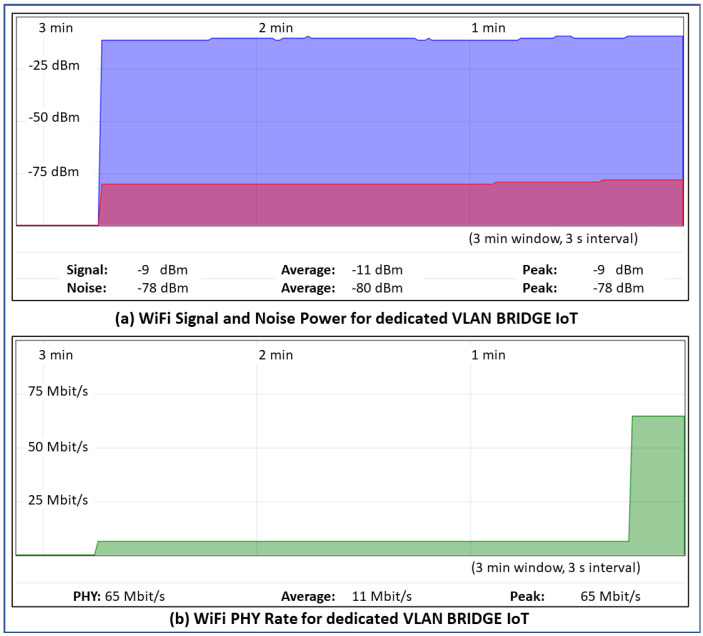
WiFi Information for dedicated VLAN BRIDGE IoT.

The router with OpenWrt firmware http://openwrt.org/ (accessed on 14 March 2024) allows for the easy configuration of complex networks, with support for VLANs, TRUNK, and network resource monitoring. In order to evaluate the physical performance limit of our testbed, Figure 4 displays the PhyRate of the WiFi network associated with the IoT VLAN. The peak value shown equals 65 Mbit/s, which represents the physical performance limit achievable with our tests due to the specific hardware. Furthermore, the same figure shows the absence of interference on the channel.

## 7. Experimental Results

**Figure 5:**
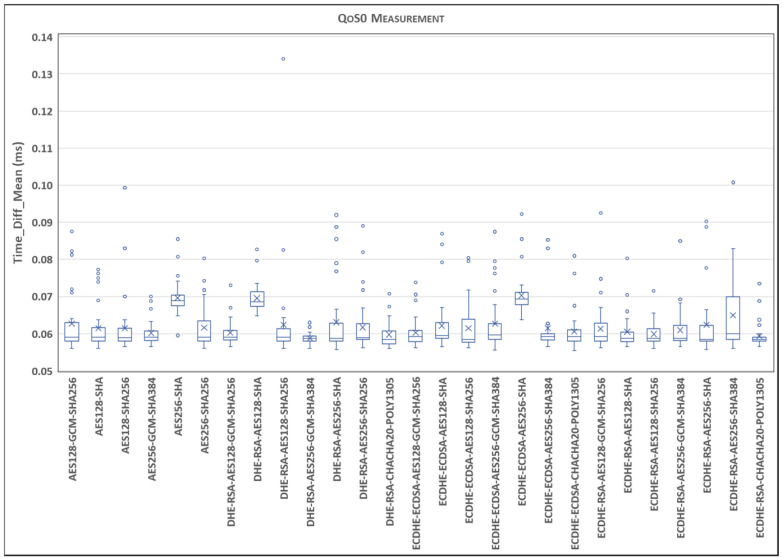
Statistical distribution of delays for TLSv1.2 cipher suites and QoS0 level on Ethernet link and MQTT V3.11.

This section presents our experimental results. According to the procedure described in Section 5, Figure 5 represents the statistical distribution of the delays for the TLSv1.2 cipher suites and QoS0 level on the Ethernet link and MQTT V3.11, comparing Qos0, QoS1, and Qos2 using a boxplot representation.

Each boxplot displays the lower and upper box limits, representing the 25th and 75th percentiles. Additionally, the lower and upper whiskers correspond to the 5th and 95th percentiles, while the median value, or the 50th percentile, is indicated by an ‘X’.

To capture the extent of the delay variation and provide insights into the previously presented average values, outlier values beyond the mentioned percentiles are included. It is important to note that the ordinate axis is divided to better illustrate the range of outlier values while presenting the boxplot range.

Figure 5 shows a stable range where most of the delay samples fall. In this sense, the outliers explain the observed variation in the average delay. As can be seen, there are few cipher suites whose delay is greater than the 95th percentile. However, Figure 5 clearly highlights that larger delays occur in the case of cipher suites: AES256-SHA, DHE-RSA-AES128-SHA, ECDHE-ECDSA-AES256-SHA. It appears that all SHAs with base AES128/256 show a larger delay, probably because they are older and have a longer negotiation time with respect to the others. Another interesting case is the cipher suite ECDHE-RSA-CHACHA20-POLY1305, which shows higher variability in terms of the interquartile range, although it is a newer cipher suite with a smaller negotiation time.

**Figure 6:**
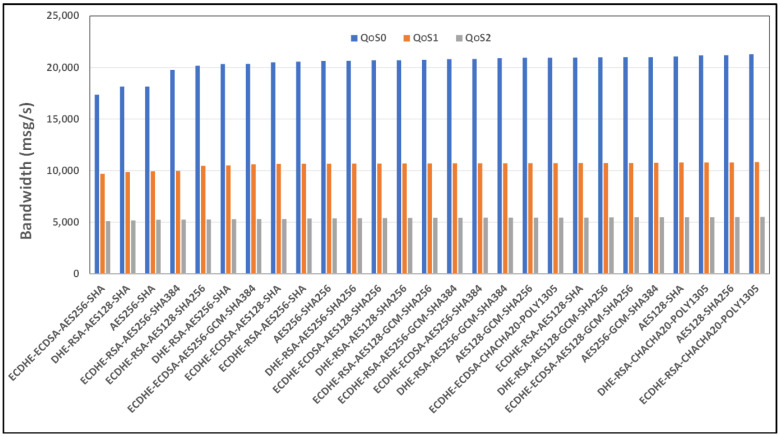
Benchmark of bandwidth for TLSv1.2 cipher suites and all QoS levels on Ethernet link and MQTT V3.11.

**Figure 7:**
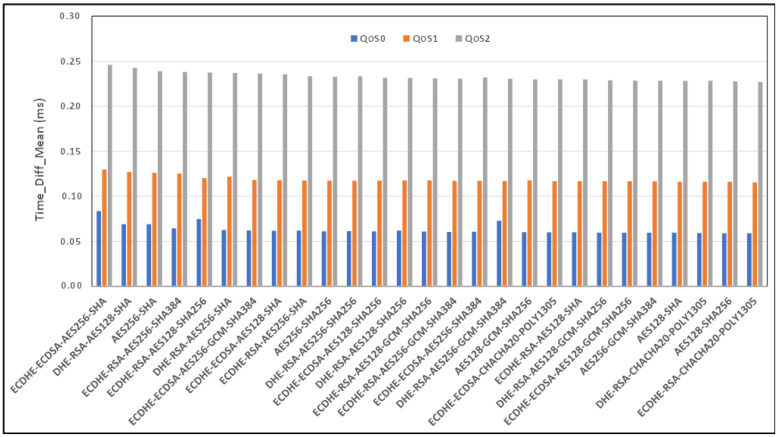
Benchmark of mean time (in milliseconds) for TLSv1.2 cipher suites and all QoS levels on Ethernet link and MQTT V3.11.

Figure 6 and Figure 7, respectively, show the comparison in terms of the bandwidth and mean time of the expensive cipher suite scenarios analyzed, considering the case of the security restrictions related to the TLS version and considering an Ethernet link connection type. As seen from Figure 6, ECDHE-RSA-CHACHA20-POLY1305 as a cipher suite appears to be the best, while ECDHE-ECDSA-AES256-SHA is the worst.

Figure 5 shows the case of the Ethernet link, which deploys an MQTT 3.x connection over TLS 1.2. Several works have already dealt with this analysis. We aimed to focus on more significant mobile IoT devices. The results obtained considering the various combinations of the MQTT and TLS protocol versions are shown below.

**Figure 8:**
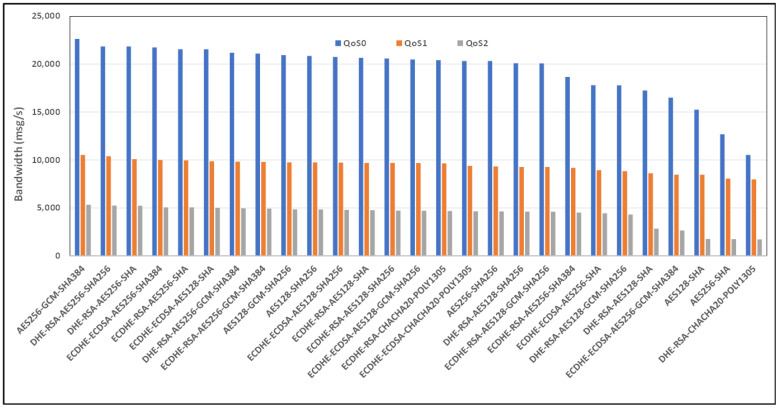
Benchmark of bandwidth for TLSv1.2 cipher suites and all QoS levels on WiFi link and MQTT V3.11.

**Figure 9:**
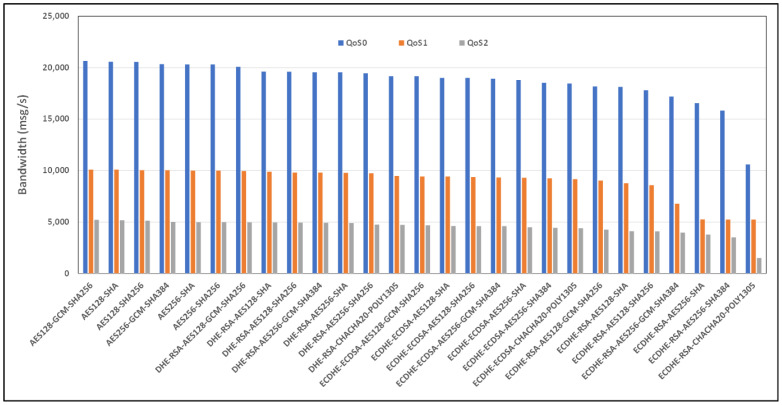
Benchmark of bandwidth for TLSv1.2 cipher suites and all QoS levels on WiFi link and MQTT V5.0.

Figure 8 and Figure 9 show the average bandwidth used during the benchmark, with cipher suites belonging to the TLS 1.2 family and MQTT versions 3.x and 5.0 with WiFi links, respectively. We can see that there are performance variations between the two protocol versions. In some cases, introducing new features may lead to an increased message transport overhead, which could negatively impact the bandwidth performance compared to the previous version of the protocol. The actual performance will also depend on the specific implementation of the MQTT protocol used by the clients and brokers. Some implementations may further optimize the bandwidth usage, while others may perform less efficiently. If we observe, for example, the performance of the AE128-SHA protocol in the MQTT 3.11 version, it appears to have very poor performance, while the MQTT 5.0 protocol version appears to have the best performance.

**Figure 10:**
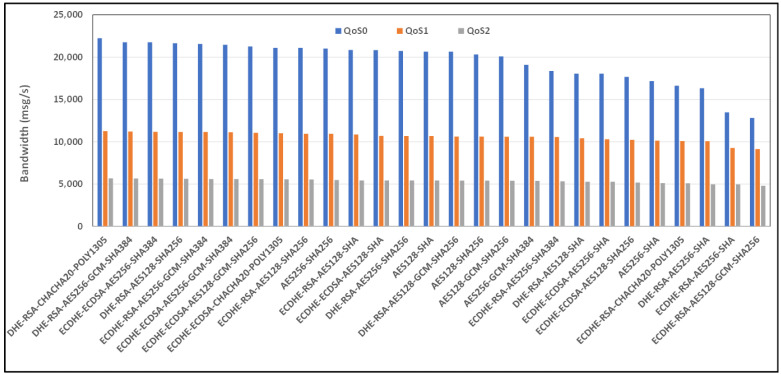
Benchmark of bandwidth for TLSv1.2 cipher suites and all QoS levels on 4G link and MQTT V3.11.

**Figure 11:**
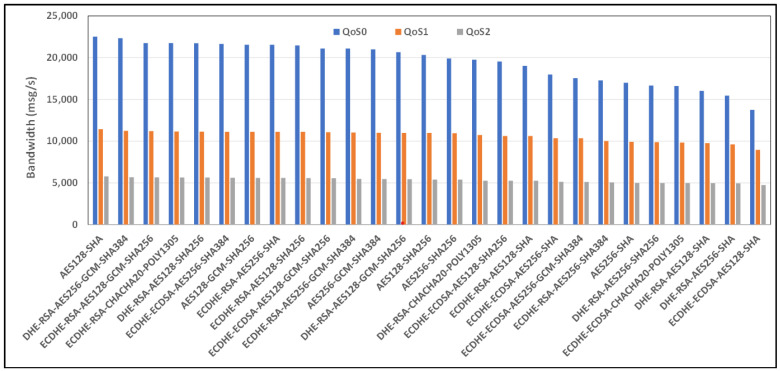
Benchmark of bandwidth for TLSv1.2 cipher suites and all QoS levels on 4G link and MQTT V5.0.

Figure 10 and Figure 11 show the average bandwidth used during the benchmark, with cipher suites belonging to the TLS 1.2 family and MQTT versions 3.x and 5.0 with 4G/LTE Links, respectively. Behavior similar to the results produced for the WiFi link can be found for the 4G link. Here, too, the performance of AE128-SHA is significantly different depending on the version of the MQTT protocol that is used. The bandwidth performance will also depend on the network environment conditions, including the latency, available bandwidth, network congestion, etc. These factors can vary significantly and impact MQTT’s bandwidth performance, especially considering 4G connections.

**Figure 12:**
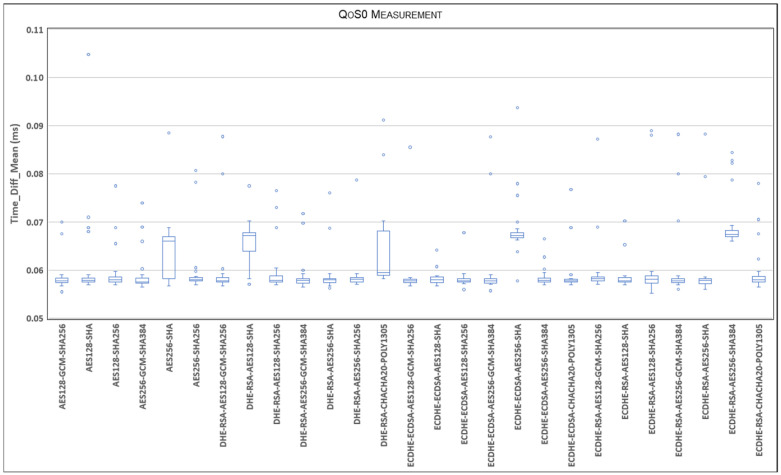
Statistical distribution of delays for TLSv1.2 cipher suites and QoS0 level on 4G link and MQTT V3.

Figure 12 shows the statistical distribution of the delays for the TLSv1.2 cipher suites and QoS0 level on the 4G link and MQTT V3.

**Figure 13:**
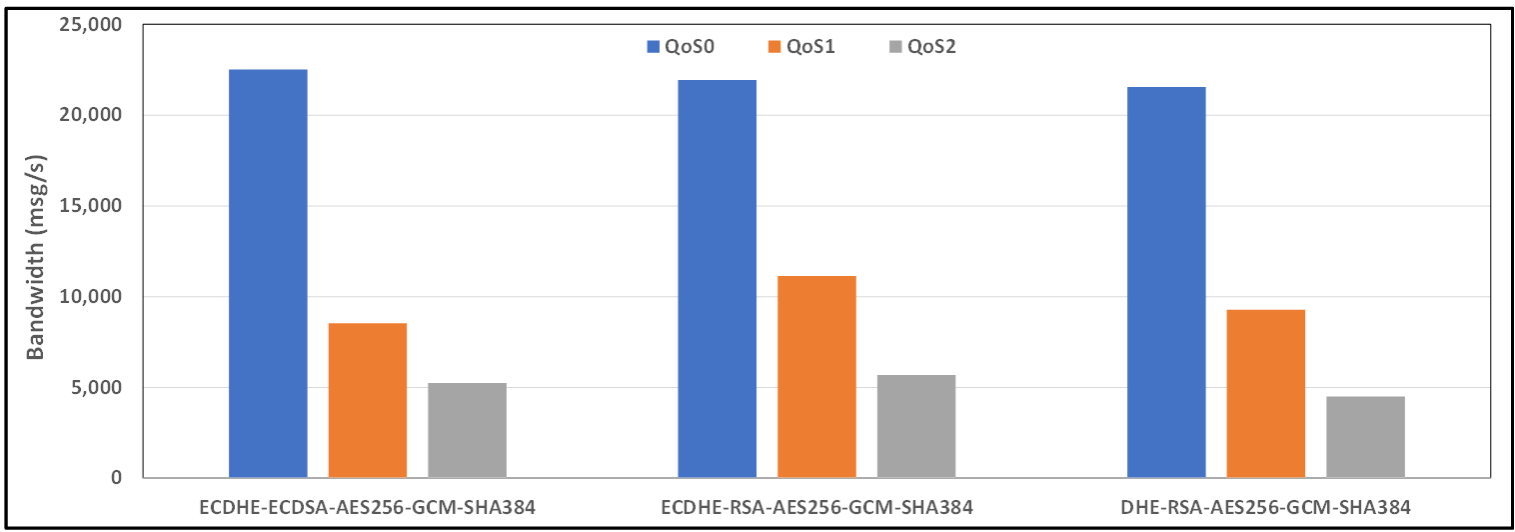
Benchmark of bandwidth for TLSv1.3 cipher suites and all QoS levels on WiFi link and MQTT V3.11.

**Figure 14:**
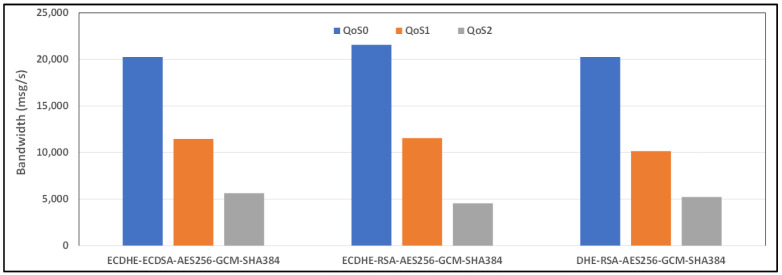
Benchmark of bandwidth for TLSv1.3 cipher suites and all QoS levels on WiFi link and MQTT V5.0.

Figure 13 and Figure 14 show the average bandwidth used during the benchmark, with cipher suites belonging to the TLSv1.3 family and MQTT versions 3.x and 5.0 with WiFi links, respectively.

**Figure 15:**
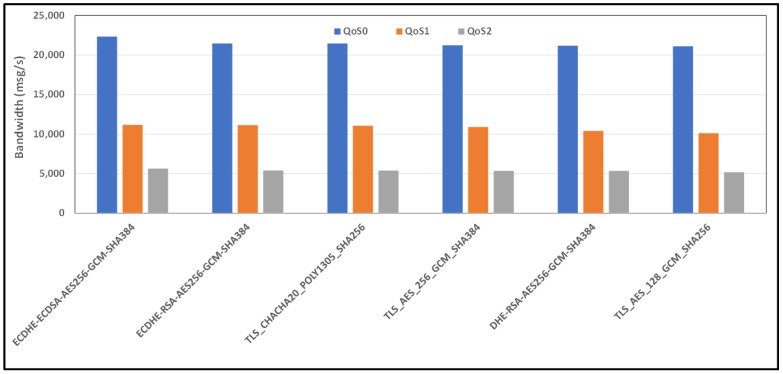
Benchmark of bandwidth for TLSv1.3 cipher suites and all QoS levels on 4G link and MQTT V3.11.

**Figure 16:**
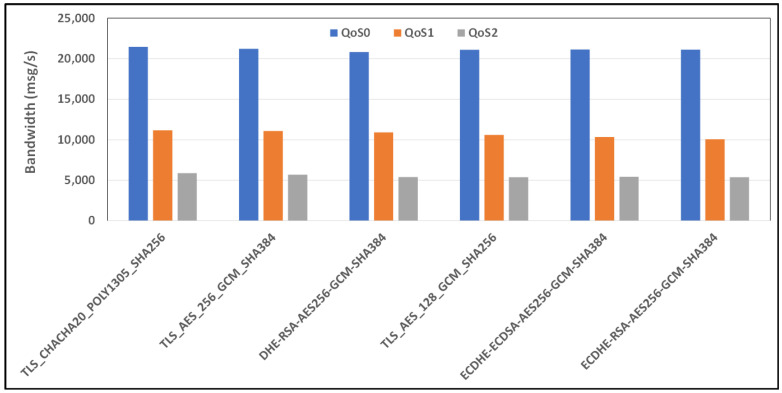
Benchmark of bandwidth for TLSv1.3 cipher suites and all QoS levels on 4G link and MQTT V5.0.

Figure 15 and Figure 16 show the average bandwidth used during the benchmark, with cipher suites belonging to the TLSv1.3 family and MQTT versions 3.x and 5.0 with 4G/LTE links, respectively.

If the family of cipher suites is changed from the TLSv1.2 algorithms to the TLSv1.3 family, we notice that when the version of the MQTT protocol changes, we do not observe significant differences in performance, as opposed to the results with the TLSv1.2 family of cipher suites.

**Figure 17:**
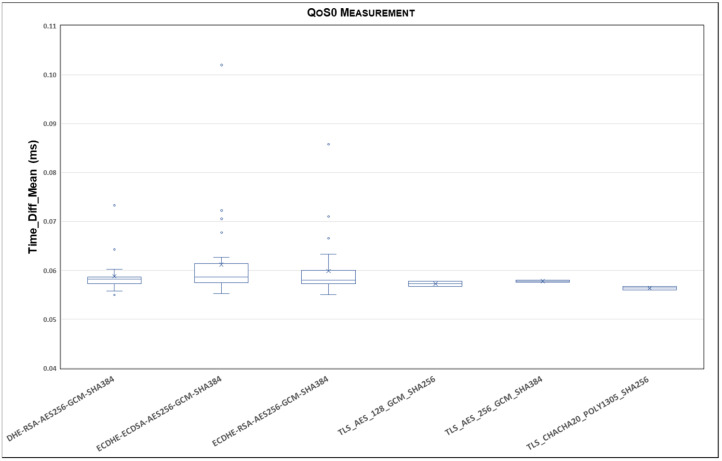
Statistical distribution of delays for TLSv1.3 cipher suites and QoS0 level on 4G link and MQTT V5.

Figure 17 shows the statistical distribution of the delays for the TLSv1.3 cipher suites and QoS0 level on the 4G link and MQTT V5.

A summary of the results of the experiments in this study is as follows.

MQTT over TLS 1.2 advantages.

- Widespread Support: TLS 1.2 is widely supported across various platforms and devices, ensuring compatibility with a wide range of MQTT implementations.
- Established Security: TLS 1.2 has been in use for many years and is well understood by developers and security experts, providing robust encryption and security for MQTT communication.
- Mature Ecosystem: With its long-standing presence in the industry, TLS 1.2 benefits from a mature ecosystem of tools, libraries, and best practices for implementation and management.

MQTT over TLS 1.2 disadvantages.

- Limited Security Features: TLS 1.2 may lack some of the advanced security features and enhancements found in newer versions, potentially exposing MQTT communication to certain vulnerabilities.
- Performance Overhead: TLS 1.2 may introduce a higher performance overhead compared to newer TLS versions, impacting the speed and efficiency of MQTT communication, particularly in resource-constrained environments.
- Potential Vulnerabilities: While TLS 1.2 provides a strong level of security, it may still be susceptible to certain known vulnerabilities or attacks, necessitating careful configuration and management to mitigate risks.

MQTT over TLS 1.3 advantages.

- Improved Security: TLS 1.3 introduces several security enhancements and performance improvements over TLS 1.2, including stronger encryption algorithms, a streamlined handshake process, and better resistance to certain types of attacks.
- Reduced Latency: TLS 1.3 reduces the latency associated with establishing a secure connection, leading to faster and more responsive MQTT communication, which is particularly beneficial for real-time applications.
- Forward Secrecy: TLS 1.3 mandates forward secrecy by default, ensuring that past communications cannot be decrypted even if the server’s private key is compromised, enhancing the overall security and confidentiality.

MQTT over TLS 1.3 disadvantages.

- Limited Adoption: Despite its advantages, TLS 1.3 may not be as widely supported as TLS 1.2, potentially causing compatibility issues with certain MQTT implementations or requiring updates to existing infrastructure.
- Complexity: Implementing and managing TLS 1.3 may require additional expertise and resources compared to TLS 1.2, as it introduces new features and protocol changes that may be unfamiliar to developers and administrators.
- Interoperability Challenges: In heterogeneous environments with a mixture of TLS 1.2 and TLS 1.3 implementations, interoperability issues may arise, necessitating careful planning and coordination to ensure seamless communication between MQTT clients and brokers.

## 8. Discussion and Conclusions

This section summarizes the paper with a discussion and conclusions.

The VLAN-based security approach for IoT networks offers both robust data security and high performance. The comprehensive evaluation and benchmarking of cipher suites and QoS levels provide insights into the optimization of IoT deployments, while data confidentiality measures address sensitive areas’ unique security needs.

Wireless technologies are undoubtedly beneficial in many scenarios, especially in external environments where connectivity is limited, such as rural or mountainous areas. Wireless connections are also useful when managing encrypted connections that cover extensive sensor networks, even when these networks consist of different types of sensors. It is possible to directly interconnect routers as active network members using protocols such as Zigbee local networks interfaced via Raspberry Pi gateways or LoRa/LoRaWAN networks, which can also be connected to LTE/4G/5G networks. This requires specific hardware and the installation of dedicated software.

This firmware is a secure option for the creation of IoT networks, even with low-cost devices. Our first objective was to identify the optimal triples (QoS, cipher suite, TLS version) that ensure the highest possible data transmission efficiency on constrained devices. We evaluated the performance of each triple for every type of QoS, cipher suite, and TLS version implemented on two specific constrained devices: the TP-LINK OpenWrt router and two Raspberry Pi 4s.

Based on the experiments conducted, the algorithms that provide the best data transmission efficiency are listed in Table 2. This table summarizes the work and provides an overview of the results obtained. In particular, using a 1 Gb Ethernet connection, the best and worst cipher suites are ECDHE-RSA-CHACHA20-POLY1305 and ECDHE-ECDSA-AES256-SHA, respectively.

Regarding 2.4 GHZ WiFi, the best and worst cipher suites are AES256-GCM-SHA384 and DHE-RSA-CHACHA20-POLY1305. Both of these values were obtained considering an MQTT version equal to 3.11 and a TLS version equal to 1.2. Finally, for the 4G/LTE area, the best and worst cipher suites are AES128-SHA with TLSv1.2 and MQTT 5.0, and TLS-AES-128-GCM-SHA256 with MQTT 3.11 and TLSv1.3. In this case, the analysis of the MQTT versions on 4G/LTE-type connections was carried out for both the 3.1x version and the 5.x version of the protocol, precisely to verify the performance achievable with the less controllable communication vector (provided by the ICT provider and not easily optimized).

Regarding our work’s second objective, the cipher suites that guarantee the best compatibility with current devices from the experiments carried out appear to be those of the TLSv1.2 family, transparently accepted by all clients tested. At the same time, TLSv1.3 is managed natively only by mqttx and hive-mqtt-cli.

As regards the third objective, the tested clients all allow the use of versions 3.1, 3.11, and 5.0 of MQTT. Still, mosquitto-clients can only negotiate TLSv1.2 and TLSv1.3 but only uses the TLSv1.2 cipher suites and cannot communicate directly via WebSocket. Mqttx and mqtt-cli, on the other hand, easily negotiate any cipher suite, TLS family, and MQTT version.

Future work could involve further analyses using different types of MQTT brokers (EMQX, RabbitMQ, NanoMQ, VernMQ) configured in increasingly complex topologies.

TLS 1.2 may be considered more suitable for constrained hardware in some cases, primarily due to its simpler cryptographic operations and broader support among existing IoT devices and platforms.

Overall, TLS 1.3 offers significant advantages in securing communication within IoT environments. It addresses the unique challenges posed by resource-constrained devices, dynamic network conditions, and evolving security threats. Its latency, security, simplicity, and performance improvements make it well suited to safeguard the integrity, confidentiality, and availability of IoT systems and data.

In summary, while MQTT over TLS 1.2 offers widespread support and a mature ecosystem, MQTT over TLS 1.3 provides improved security and reduced latency. The choice between the two depends on factors such as security requirements, performance considerations, and compatibility with existing infrastructure, as reported in [41,48,49].

## Figures and Tables

**Table 1:** OpenSSL cipher suites for MQTT testbeds.

TLSv1.2	TLSv1.3
ECDHE-ECDSA-AES128-GCM-SHA256	TLS_AES_128_GCM_SHA256
ECDHE-RSA-AES128-GCM-SHA256	TLS_AES_256_GCM_SHA384
ECDHE-ECDSA-AES256-GCM-SHA384	TLS_CHACHA20_POLY1305_SHA256
ECDHE-RSA-AES256-GCM-SHA384	–
ECDHE-ECDSA-CHACHA20-POLY1305	–
ECDHE-RSA-CHACHA20-POLY1305	–
DHE-RSA-AES128-GCM-SHA256	–
DHE-RSA-AES256-GCM-SHA384	–

**Table 2:** Results: The table shows the worst and best results in terms of the measured bandwidth (msg/s), taking QoS0 values as a reference.

COMMUNICATION MEDIUM	CIPHER SUITE	MQTT	TLS	WORST	BEST
**GIGABIT ETHERNET**	ECDHE-RSA-CHACHA20-POLY1305	V3.11	V1.2		21,295
**GIGABIT ETHERNET**	ECDHE-ECDSA-AES256-SHA	V3.11	V1.2	17,353	
**WiFi-2.4**	AES256-GCM-SHA384	V3.11	V1.2		22,623
**WiFi-2.4**	DHE-RSA-CHACHA20-POLY1305	V3.11	V1.2	10,527	
**WiFi-2.4**	ECDHE-ECDSA-AES256-GCM-SHA384	V3.11	V1.3		22,525
**WiFi-2.4**	DHE-RSA-AES256-GCM-SHA384	V3.11	V1.3	21,553	
**WiFi-2.4**	AES128-GCM-SHA256	V5.0	V1.2		20,661
**WiFi-2.4**	ECDHE-RSA-CHACHA20-POLY1305	V5.0	V1.2	10,597	
**WiFi-2.4**	ECDHE-RSA-AES256-GCM-SHA384	V5.0	V1.3		21,553
**WiFi-2.4**	DHE-RSA-AES256-GCM-SHA384	V5.0	V1.3	20,242	
**4G-LTE**	DHE-RSA-CHACHA20-POLY1305	V3.11	V1.2		22,239
**4G-LTE**	ECDHE-RSA-AES128-GCM-SHA256	V3.11	V1.2	12,819	
**4G-LTE**	AES128-SHA	V5.0	V1.2		22,526
**4G-LTE**	ECDHE-ECDSA-AES128-SHA	V5.0	V1.2	13,743	
**4G-LTE**	ECDHE-ECDSA-AES256-GCM-SHA384	V3.11	V1.3		22,333
**4G-LTE**	TLS-AES-128-GCM-SHA256	V3.11	V1.3	5376	
**4G-LTE**	TLS-CHACHA20-POLY1305-SHA256	V5.0	V1.3		21,461
**4G-LTE**	ECDHE-RSA-AES256-GCM-SHA384	V5.0	V1.3	21,113	

## Data Availability

Data are contained within the article.
